# The first live term birth following uterus transplantation in Australia

**DOI:** 10.5694/mja2.52682

**Published:** 2025-05-23

**Authors:** Rebecca Deans, Brigitte Gerstl, Antonia W Shand, Sarah Lyons, Aaron Budden, Helen L Barrett, Grant Luxton, Mangalee Fernando, Kenneth Yong, Karen Keung, Kaushalya Arulpragasam, Henry Pleass, King Man Wan, Eva Kehag, Jana‐Emily Pittman, Mianna Lotz, Maria Fenn, Erin Nesbitt‐Hawes, Lily Byun, Katrina Tang, Mats Brannstrom, Jason Abbott

**Affiliations:** ^1^ Royal Hospital for Women Sydney NSW; ^2^ The University of New South Wales Sydney NSW; ^3^ Prince of Wales Private Hospital Sydney NSW; ^4^ Children’s Hospital at Westmead Clinical School, the University of Sydney Sydney NSW; ^5^ Women’s Reproductive Care Coffs Harbour NSW; ^6^ Prince of Wales Hospital Sydney NSW; ^7^ Westmead Hospital Sydney NSW; ^8^ The University of Sydney Sydney NSW; ^9^ Macquarie University Sydney NSW; ^10^ NSW Health Pathology Sydney NSW; ^11^ Sahlgrenska Academy, University of Gothenburg Gothenburg Sweden

**Keywords:** Transplantation, Pregnancy, Immunosuppression, Birth weight, Infertility

## Abstract

**Objective:**

To report the first live birth following uterus transplantation in Australia.

**Study design:**

Case report.

**Setting, participant:**

The first participant in the uterus transplantation research study program at the Royal Hospital for Women, the Prince of Wales Hospital, and Westmead Hospital in Sydney.

**Main outcome measures:**

Clinical course after uterus transplantation; course of the subsequent pregnancy until delivery.

**Results:**

The immunosuppression regimen following uterus transplantation on 10 January 2023 was similar to that used for low immunologic risk kidney transplantation. It included induction therapy (basiliximab on days 0 and 4, methylprednisolone on days 0 and 1), followed by maintenance therapy with oral tacrolimus, prednisolone, and mycophenolate mofetil (MMF). The prednisolone dose was steadily tapered over twelve weeks to a low maintenance dose (from 25 mg to 5 mg daily); MMF was replaced with azathioprine during week 9, and tacrolimus was continued throughout the pregnancy. There was no evidence of rejection. A frozen grade 1 blastocyst was transferred during a natural ovulatory cycle 101 days (fifteen weeks) after transplantation; clinical pregnancy was successfully initiated. The woman developed gestational diabetes at 20 weeks and was treated with insulin. A healthy boy was born by planned caesarean delivery at 37 weeks; he weighed 2990 g, with Apgar scores of 7 at one minute and 9 at five minutes. Intrapartum haemorrhage (estimated 2500 mL) led to iron infusion after delivery. The woman and her infant were discharged from the hospital five days after the birth. The infant was breastfed, but the woman experienced recurrent episodes of mastitis that were managed with oral antibiotics, and intravenous antibiotics during two hospital admissions. Eight weeks after birth she commenced weaning the infant. Neither the woman nor her infant experienced serious complications.

**Conclusion:**

The first live birth following uterus transplantation in Australia indicates that the procedure could be adopted here as an assisted reproductive technology for women with uterine factor infertility.

**Trial registration:**

Australian and New Zealand Clinical Trials registry, ACTRN12622000917730.

**The known**: In Australia, the options for women with uterine factor infertility who wish to have children are adoption and surrogacy. Both options are limited by legal, availability, and ethical barriers.

**The new**: The first live birth after uterus transplantation in Australia confirms that it could be a solution for women with uterine factor infertility who wish to have children with whom they are biologically related.

**The implications**: Uterus transplantation is a new reproduction alternative for women with uterine factor infertility. Further research and regulation are important for refining surgical procedures and assessing surgical risks, immunosuppression effects, obstetric complications, costs, and benefits, as well as the long term medical and psychological effects.

Australian women with uterine factor infertility can currently become parents only through surrogacy or adoption. Barriers to these options include legal and religious restrictions, limited availability, and the prohibition of compensated surrogacy.^1^, ^2^ Ethical concerns about transnational surrogacy include the possibility of exploitation and the risks of medical tourism.^3^, ^4^

One in 500 women of reproductive age have an absent or malfunctioning uterus,^5^ which may be caused by the congenital absence of the uterus, Müllerian duct anomalies, or acquired conditions. Uterus transplantation (UTx) is a promising alternative for women with uterine factor infertility who wish to experience pregnancy and childbirth. More than 80 UTx procedures around the world, with living or deceased donors, have resulted in about 40 live births.^6^, ^7^, ^8^, ^9^, ^10^ In 2014, the first live birth following UTx took place in Sweden, after directed donation by a living donor.^1^

Major advances in reproductive technologies have been achieved in Australia, particularly in in vitro fertilisation (IVF); the third baby in the world conceived by IVF was born in Australia in 1980,^11^ and the first donor egg and first frozen embryo pregnancy was undertaken here in 1983,^12^ illustrating the local commitment to innovative reproductive solutions.^12^, ^13^ The Australian regulatory framework, its health care structure, and strong research–clinical collaboration ensure high standards for reproductive technologies.

As a novel procedure, UTx raises substantial ethical questions. Because live uterus donors undergo major surgery that entails risks of complications and long term health effects, fully informed consent is required, free from coercion, especially within families.^1^, ^14^ As the long term outcomes for both donors and recipients, as well as for the children born from transplanted uteri, are unknown, further research and monitoring is essential.^15^ The allocation of substantial medical and financial resources to UTx, potentially exacerbating health care inequalities, raises questions about equity and access.^16^ The psychological impact of UTx on both donors and recipients, including the possible emotional strain and pressure of procedural failure, make comprehensive support essential.^17^ Finally, immunosuppressive therapy for recipients increases risks during future pregnancies. Addressing these challenges is essential for the safe and ethical development of UTx.^18^

UTx is not permanent; the timing of removal of the uterus from the recipient differs between transplantation centres, but most protocols require removal after one or two successful pregnancies, or within five years.^17^ Since the establishment of proof of concept, the justification for this non‐life saving procedure is a core question.^7^ We report the first birth in Australia after UTx, undertaken in collaboration with the Swedish team who performed the first successful UTx procedure, and discuss the rationale for using this procedure in Australia.

## Methods

Six women with uterine factor infertility participated in our clinical trial, undertaken at the Royal Hospital for Women, the Prince of Wales Hospital, and Westmead Hospital in Sydney.^18^, ^19^ Three UTx procedures have been performed, using uteri from living donors; two recipients have given birth to live babies, and an embryo transfer is planned for a third woman. In this article, we report the first UTx procedure, undertaken at the Royal Hospital for Women on 10 January 2023. The clinical trial was approved by the Western Sydney Local Health District human research ethics committee (2019/ETH13038), and was prospectively registered with the Australian and New Zealand Clinical Trials registry (27 June 2022; ACTRN12622000917730). The woman who received the transplant provided written informed consent for the publication of her case.

## Results

Following extensive screening, a 31‐year‐old woman with uterine factor infertility (post partum hysterectomy after a massive haemorrhage) was the first person enrolled in the trial, having met all eligibility criteria.^18^ Her blood group was A+, she did not smoke, and her body mass index was 25.7 kg/m^2^; the only medication she was currently using was the antidepressant fluoxetine (20 mg daily). The donor was the recipient’s 53‐year‐old mother; her blood group was also A+, she had twice given birth at term (vaginal delivery) and had not undergone menopause, did not smoke, and her BMI was 26.8 kg/m^2^. Anti‐human leukocyte antigen (HLA) mismatch between the donor and recipient was 3/6; no anti‐HLA donor‐specific antibodies were detected. Flow cytometry did not identify T‐ or B‐cell crossmatches. Both the donor and recipient were IgG‐negative for cytomegalovirus (CMV) and IgG‐positive for Epstein–Barr virus (EBV).

### Immunosuppression

The recipient was prescribed the usual induction immunosuppression for low immunologic risk kidney transplantation: basiliximab (two doses, day 0 and day 4) and intravenous methylprednisolone (during the procedure on day 0, and 24 hours later on day 1). Maintenance immunosuppression comprised twice daily oral tacrolimus (trough target levels, assessed by automated chemiluminescent immunoassay, Abbott Diagnostics: 9–11 ng/mL immediately after transplantation; 8–10 ng/mL during weeks 9 to 12; 5–8 ng/mL during pregnancy until delivery), daily prednisolone (initially 25 mg daily, reduced to 5 mg daily by week 13), and twice daily mycophenolate mofetil (MMF; initially 1 g twice daily, reduced to 750 mg twice daily from week 3). At week 9, MMF was replaced by azathioprine (2 mg/kg daily; absolute dose: 150 mg daily) after confirmation of normal thiopurine methyltransferase activity, because of its superior safety profile in pregnant women (Box). Urine cultures were undertaken when indicated (ie, if the woman had lower urinary tract symptoms or infection was otherwise suspected). Urine albumin:creatinine ratio was assessed monthly after UTx. Anti‐HLA donor‐specific antibodies were assessed prior to and one and four weeks after UTx, and as indicated. Prophylaxis against viral (valaciclovir), *Candida* (fluconazole, followed by nystatin drops), and *Pneumocystis jirovecii* infections (80 mg trimethoprim/400 mg sulfamethoxazole) was initiated on the day of surgery) and discontinued prior to embryo transfer. The woman was reviewed three times a week by the Prince of Wales Hospital transplantation team during the first month, primarily for monitoring and managing immunosuppression, reduced to fortnightly follow‐up by the end of the third month after surgery. At each visit, pathology (haematology, serum biochemistry, including renal function, tacrolimus trough levels, urine microscopy and culture, proteinuria) and clinical parameters (weight, blood pressure) were assessed. Side‐effects of immunosuppression included viral gastrointestinal and upper respiratory tract infections.

Box 1 **Immunosuppression schedule after uterus transplantation**

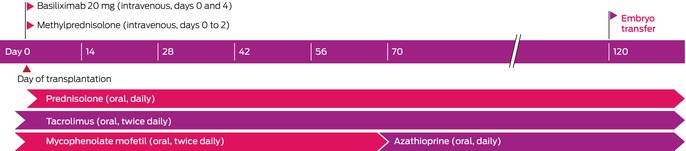

### Monitoring of the graft

Gynaecological assessments were undertaken and transplanted cervix biopsy samples collected to monitor signs of graft failure or rejection, weekly during the first month, fortnightly during the second month, and then monthly until the start of the pregnancy, during which assessments were conducted each trimester. A total of nine cervical biopsy samples were collected between transplantation and the birth of the infant; they were histologically assessed for organ rejection using a grading system developed by the Swedish team.^20^

Endometrial thickness and uterine vascularity were monitored by ultrasound and Doppler imaging on the first day after surgery and at the same time as cervix biopsy sample collection. The abdominal probe was positioned superior to the inguinal ligament to assess arterial flow velocity waveforms for the two uterine arteries and four venous outlets.

### Antenatal care

A frozen grade 1 blastocyst^21^ was transferred during a natural ovulatory cycle 101 days (fifteen weeks) after UTx and six weeks after cessation of MMF treatment, and clinical pregnancy was successfully initiated 112 days after UTx. An episode of early pregnancy bleeding was detected at seven weeks; ultrasound scanning indicated that the fetal heart rate was good, and no cause for haemorrhage was identified. Fortnightly visits to review blood pressure and for urinalysis (microscopy, proteinuria) were scheduled for weeks 12–34, followed by weekly visits until delivery. First trimester screening for pregnancy‐associated plasma protein A (pappalysin‐1), human chorionic gonadotropin (β‐hCG), placental growth factor (PGF), and inflammatory markers were undertaken during weeks 11 or 12, as were iron store assessments, liver and renal function testing, and oral glucose tolerance testing (OGTT). During weeks 12 or 13^+6^, nuchal translucency scanning for chromosomal abnormalities, pre‐eclampsia screening, and testing for CMV IgM and IgG were undertaken. Cervical length was measured during gestation weeks 16 to 34. At 20 weeks, gestational diabetes mellitus was diagnosed after OGTT; it was presumed to be steroid‐induced, and treatment with long acting insulin was commenced and adjusted to a maximum dose of 26 U per day. A detailed fetal anatomical scan was conducted at 20 weeks; iron studies, full blood count, urea, electrolytes, and creatinine assessments, liver function tests, and antibody screening were conducted at 28 and 34 weeks.

Ultrasound assessments of graft function were combined with assessments of the growth and wellbeing of the fetus. The pulsatility index of the uterine arteries and umbilical artery, and fetal growth were compared with Australian normal reference values, confirming normal fetal growth and placental function. Immunosuppressive treatment with tacrolimus, azathioprine, and prednisolone were continued during the pregnancy. As the target tacrolimus level was reduced over time, gradually adjusted to 60% of the pre‐pregnancy dose (target level, 5–7 ng/mL). At 35 weeks of pregnancy, the woman was admitted to hospital with headache and nausea, but her blood pressure was normal and she was discharged within 24 hours.

### Delivery of the baby

An elective caesarean delivery was performed at 37 weeks’ gestation; the woman received combined spinal–epidural anaesthesia and prophylactic tranexamic acid (1 g intravenous). The fetus was in the cephalic position, and was delivered through a midline laparotomy incision along the existing scar (below the navel to the pubic bone) and a lower segment uterine incision, completed thirteen minutes after the initial skin incision. The umbilical cord was normal (three vessels, no signs of inflammation). After delivery, the uterus exhibited effective contraction in response to 10 IU intramuscular oxytocin and 40 IU infused oxytocin. The uterine incision was sutured using two layer closure. The estimated blood loss was 2.5 L, predominantly from the left uterine venous complex, where additional haemostatic sutures were placed; no blood products were transfused. The pre‐delivery haemoglobin level was 109 g/L; as the level was 77 g/L after delivery, iron infusion was initiated, and at discharge the level had reached 84 g/L.

### Neonatal outcomes

The boy weighed 2990 g at birth, was 49 cm long, and had a head circumference of 34 cm. Apgar scores were 7 at one minute and 9 at five minutes; the umbilical artery pH was 7.22, the venous pH 7.31. Following delivery, the infant showed signs of mildly increased respiratory effort, resolved by airway suction and oxygen therapy by continuous positive airway pressure for ten minutes after delivery, but he did not need special care or admission to the intensive care nursery. The infant was breastfed and developed mild jaundice on day 3, treated in hospital with light therapy.

### Post‐natal care

The woman was discharged from hospital five days after giving birth. Immunosuppression was continued after discharge; enoxaparin sodium was prescribed for prophylactic anticoagulation for six weeks (40 mg in 0.4 mL daily). The woman was given analgesics and laxatives to support recovery. Three days after discharge, she developed mastitis and contacted the uterus transplantation team at the Royal Hospital for Women; she was referred to her local hospital, where she was treated with oral and intravenous antibiotics. Following two further episodes of mastitis, she commenced weaning eight weeks after the birth and completely ceased breastfeeding twelve weeks after giving birth. Immunosuppression and antithrombotic treatment continued. The first post partum cervix biopsy, performed six weeks after delivery, did not find any signs of organ rejection. However, subsequent cervix biopsies on 22 May and 3 June 2024 found signs of inflammation and non‐specific rejection. Donor‐specific antibodies were detected in blood tests on 8 May and 1 July 2024. The woman was treated for organ rejection (as an outpatient) with intravenous methylprednisolone (500 mg daily) for three consecutive days (2–4 July 2024). Subsequent biopsies found signs of inflammatory changes consistent with ongoing rejection. After considering further treatment and its side‐effects, she elected to undergo an explant hysterectomy. It was performed laparoscopically on 12 September 2024, and took 2.25 hours. Following surgery, immunosuppression was reduced (2 mg prednisolone monthly), and was ceased on 10 January 2025.

### Current status of the infant

The infant is now fifteen months old and meeting all developmental milestones.

## Discussion

The first live birth after the first UTx procedure undertaken in Australia illustrates the potential utility of UTx for local women with uterine factor infertility. We report the first live birth after UTx in southeast Asia or Oceania. For the first pregnancy following UTx, in Sweden, graft function had been stable for at least one year before the pregnancy;^22^ embryo transfer times as short as 183 days after transplantation have been reported in the United States.^15^, ^23^ Aiming to minimise the duration of immunosuppression and its potential side‐effects, including renal impairment and malignancy, we decided that the earliest point for safe embryo transfer was after MMF washout, given its potentially fetotoxic effects. Embryo transfer was undertaken on day 101, resulting in clinical pregnancy 112 days after UTx. This approach not only reduces the duration of immunosuppression for the recipient, it also reduces their waiting time for childbirth, fulfilling the primary reason for transplantation and undergoing the costs and risks of long term immunosuppression.^15^, ^24^, ^25^ The time from UTx to delivery of a live term infant, within a single calendar year, is the shortest reported to date. The new standard for the first embryo transfer after UTx could be two to three months if no problems arise during this period.

For most UTx procedures and live births, the organs have been donated by live donors.^9^, ^26^ Early graft survival has been higher for uteri from live than deceased donors (43 of 68, 77% *v* 15 of 22 transplants, 68%),^7^, ^8^, ^9^, ^27^ as is the live birth rate, both before (28 of 56, 50% *v* 7 of 22 transplants, 32%) and after excluding early graft failures (28 of 43, 65% *v* 7 of 15 transplants, 47%).^8^, ^9^ The major reason for considering deceased donors for UTx is to avert the complications experienced by live donors. Seven of 35 live donors in the International Society of Uterus Transplantation (ISUTx) register reported complications, with three classified as Clavien–Dindo grade III or higher, requiring surgical or interventional management.^8^, ^28^ In our study, the donor recovered as expected following the donation; urinary tract infections and reduced voiding sensation had resolved within twelve months of surgery, and she has subsequently been in good health.

Clinical outcomes following solid organ transplantation in Australia are among the best in the world.^24^ To optimise the success of UTx, factors that facilitate these good outcomes are important: government‐subsidised health care for life (including physician fees, tests, medications, and hospital costs); prospective capture of outcomes data, reported to both health care providers and the public; and a strong culture of regulation, research, and clinician education. The Australian UTx research team is assisting with the development of guidelines by the Transplantation Society of Australia and New Zealand that encompass both live and deceased donor pathways, to establish a regulatory framework for the reproductive technology in Australia and New Zealand. Moreover, the UTx team contributes its findings to the ISUTx registry; as fewer than one hundred UTx procedures and 40 live births have been reported worldwide, the collection and dissemination of information from participants in the international consortium ensures the safe translation from research to clinical practice. As the risks to living UTx donors and the recipients become clearer,^29^ improving the outcomes for deceased donor organs will be the key to ensuring the success of UTx. In Australia, the number of deceased organ donors was 7.8% higher in 2022 than in 2021;^30^ considering uterus retrieval as part of multi‐organ donation would be the next step in developing a safe UTx clinical service.

The costs and benefits of UTx must be assessed before initiating a UTx clinical program in Australia. Both the pregnancies achieved during our clinical trial followed first embryo transfers. The United States consortium reported that 17 of 19 women became pregnant after the first or second embryo transfer; two required three or more transfers.^9^ There are no limitations on Medicare‐subsidised IVF cycles in Australia, and clinicians assess prognostic indicators (age, ovarian reserve) at their discretion. Women using assisted reproductive technology in Australia undergo a mean of two treatment cycles, and about 11% undergo four or more fresh or thaw cycles.^31^

The costs of repeated oocyte stimulation and embryo transfer for women with other causes of infertility and a poor prognosis for conception are similar to those for UTx. A recent analysis found that the cost for achieving a live birth with unrestricted Medicare funding was $76 759 for women aged 41 or 42 years and $436 694 for women more than 45 years old.^32^ We believe that the cost of UTx in Australia is similar to that estimated by the converted cost analysis of the first Swedish UTx series; including pre‐operative investigations, IVF, and live donor uterus transplantation and post‐procedure costs for two months, it was equivalent to $120 000.^24^ The costs associated with altruistic surrogacy in Australia range from $35 000 to $100 000;^33^ in the United States, the estimated cost for gestational carriers ranges from $100 000 to $200 000.^6^, ^9^ UTx costs have declined in recent years because of advances in surgical techniques, earlier embryo transfer, and reduced time to pregnancy. In most UTx protocols, a transplanted uterus can be sustained for two separate deliveries, but the expenses for a surrogate pregnancy recur with each subsequent attempt. These comparisons suggest that the costs per child are similar for UTx and surrogacy.^6^

Further, in surrogacy the risks of pregnancy are transferred to a third party, whereas in UTx they are borne by the intending mother.^34^ The number of altruistic surrogate women is limited, and surrogacy gestational carrier cycles comprised only 0.3% of assisted reproductive technology treatment cycles in Australia and New Zealand during 2018.^35^ As commercial surrogacy is prohibited in Australia, some women and their partners seek commercial surrogacy arrangements overseas, which are expensive and entail risks of complications.^5^, ^36^, ^37^ Adoption does not provide a genetic link between mother and child, or the possibility for women to experience the physical and emotional aspects of pregnancy; the number of children available for adoption in Australia is, in any case, limited.^38^

In contrast to kidney donation, removal of a uterus does not affect any physiologic function in the donor, although hysterectomy has inherent risks.^34^ Medicare and Australian transplantation physicians have accepted other life‐enhancing transplants, such as a hand transplantation in 2011.^39^ Pancreas transplantation is performed at three Australian centres and islet isolation at two nationally funded centres to ensure equitable access.^40^ UTx provides a life‐enhancing, temporary transplant, without many of the negative effects of long term immunosuppression that affect other vascular composite allograft transplants. As with other high cost, low volume procedures, and all transplantation, centralising UTx would facilitate the concentration of expertise, ensuring best practice. Retaining UTx in a governed public health service allows equitable access and reduces the need for medical tourism, and is aligned with the principles of the World Health Assembly for protecting people in developing countries from exploitation for organ and tissue transplantation.^41^

### Conclusion

We report the first live birth after UTx in Australia. UTx can give hope to women with uterine factor infertility, allowing them to experience both pregnancy and a biological connection with their child. In low volume, highly technical procedures, efficacy and safety depend on continuing research and international collaboration. Specialist centres that concentrate expertise will ensure best practice. Rigorous research, audit, and collaboration, supported by experienced multidisciplinary teams, is essential for developing a UTx program in Australia that meets the highest international medical and ethical standards.

## Open Access

Open access publishing facilitated by University of New South Wales, as part of the Wiley – University of New South Wales agreement via the Council of Australian University Librarians.

## Competing interests

No relevant disclosures.

## Data sharing

Our research team are committed to sharing de‐identified data from our study if required. The full compilation of uterus transplantation data will become available following completion of the six uterus transplantations in this study. Access to data can also be provided on request, subject to a data sharing agreement that ensures appropriate use and the confidentiality of all participants.

Received 16 April 2024, accepted 3 September 2024
